# Olfactory Dysfunction in a Novel Model of Prodromal Parkinson’s Disease in Adult Zebrafish

**DOI:** 10.3390/ijms26104474

**Published:** 2025-05-08

**Authors:** Nathaniel W. Vorhees, Samantha L. Groenwold, Mackenzie T. Williams, Lexus S. Putt, Nereyda Sanchez-Gama, Grace A. Stalions, Gabriella M. Taylor, Heather E. Van Dort, Erika Calvo-Ochoa

**Affiliations:** Biology Department and Neuroscience Program, Hope College, Holland, MI 49423, USA

**Keywords:** olfactory system, zebrafish, Parkinson’s disease, olfactory dysfunction, neuroinflammation, neurogenesis, dopaminergic loss

## Abstract

Olfactory dysfunction is a clinical marker of prodromal Parkinson’s disease (PD), yet the underlying mechanisms remain unclear. To explore this relationship, we developed a zebrafish model that recapitulates the olfactory impairment observed in prodromal PD without affecting motor function. We used zebrafish due to their olfactory system’s similarity to mammals and their unique nervous system regenerative capacity. By injecting 6-hydroxydopamine (6-OHDA) into the dorsal telencephalic ventricle, we observed a significant loss of dopaminergic (DA) periglomerular neurons in the olfactory bulb (OB) and retrograde degeneration of olfactory sensory neurons (OSNs) in the olfactory epithelium (OE). These alterations impaired olfactory responses to cadaverine, an aversive odorant, while responses to alanine remained intact. 6-OHDA also triggered robust neuroinflammatory responses. By 7 days post-injection, dopaminergic synapses in the OB were remodeled, OSNs in the OE appeared recovered, and neuroinflammation subsided, leading to full recovery of olfactory responses to cadaverine. These findings highlight the remarkable neuroplasticity of zebrafish and suggest that this model of olfactory dysfunction associated with dopaminergic loss could provide valuable insights into some features of early PD pathology. Understanding the interplay between dopaminergic loss and olfactory dysfunction in a highly regenerative vertebrate may inform therapeutic strategies for individuals suffering from olfactory loss.

## 1. Introduction

Parkinson’s disease (PD) is a prevalent neurodegenerative disorder characterized by severe motor impairment, cognitive decline, and increased mortality in aging populations [1,2]. Notably, over 95% of PD patients experience olfactory dysfunction, often preceding motor symptoms by several years, making it a key early clinical marker of prodromal PD [3,4,5].

The progressive and irreversible degeneration of dopaminergic (DA) neurons, particularly in the substantia nigra pars compacta, is a hallmark of PD, leading to widespread disruptions in dopaminergic neural circuits [6,7]. Among the affected regions is the olfactory bulb (OB), the primary center for olfactory processing, which contains a dense population of dopaminergic periglomerular neurons crucial for odor detection and discrimination [8,9]. Since the OB is one of the earliest structures impacted in PD [10,11], dopaminergic dysfunction within this region may contribute to the olfactory deficits that are apparent years before motor symptoms manifest.

Although olfactory impairment in PD is extensive, research on the topic remains limited, highlighting the need for further investigation. To explore this, we used zebrafish, a well-established model for PD research due to its genetic similarity to humans (~70% of genes are conserved [12]) and its remarkable capacity for nervous system repair and regeneration [13].

Broadly, there are two types of experimental strategies for achieving PD-like pathology in zebrafish. Transgenic lines are used to study the genetic underpinnings of familial PD [14,15], while neurotoxin-exposure models are used to replicate key features of sporadic PD, which accounts for ~95% of cases [15]. Among neurotoxins, the most commonly used include 6-OHDA, MPTP, rotenone, and paraquat, all of which induce mitochondrial dysfunction, oxidative stress, and neuroinflammation, ultimately leading to catecholaminergic neuronal loss [16,17,18,19].

We selected 6-OHDA for this study due to its specificity in targeting catecholaminergic neurons via uptake by the dopamine transporter, leading to localized neurodegeneration upon uptake [20]. Importantly, 6-OHDA injections allow for the targeting of specific DA populations [21,22], making it an advantageous tool for targeting DA periglomerular neurons in the OB. Despite extensive research on PD in zebrafish, the relationship between dopaminergic loss and olfactory dysfunction in relationship with PD neuropathology has not been explored.

An important consideration is that olfactory assessments in zebrafish typically rely on motor-behavioral assays, resulting in potential confounds if there are motor impairments, which is often the case with PD models. To overcome this challenge, we sought to develop a model of early PD-related olfactory dysfunction without locomotor deficits, recapitulating one of the features of the prodromal stage of the disease. We achieved this by injecting 6-OHDA into the dorsal telencephalic ventricle of adult zebrafish, selectively targeting DA periglomerular neurons in the OB. By using zebrafish, we could also explore neuroplasticity and regenerative mechanisms following dopaminergic loss normally absent in mammalian models of PD.

Here, we demonstrate that olfactory dysfunction results as a consequence of DA loss in the OB. Olfactory morphology and function recovered within seven days, underscoring the high neuroplasticity of the zebrafish olfactory system. This model of prodromal PD provides a valuable tool for investigating the early stages and progression of this neurodegenerative disease and could offer new insights into the relationship between dopaminergic loss and olfactory dysfunction.

## 2. Results

### 2.1. 6-OHDA Injections in the Dorsal Telencephalic Ventricle Target Dopaminergic Neurons in the OB Without Impairing Motor Function

**Figure 1:**
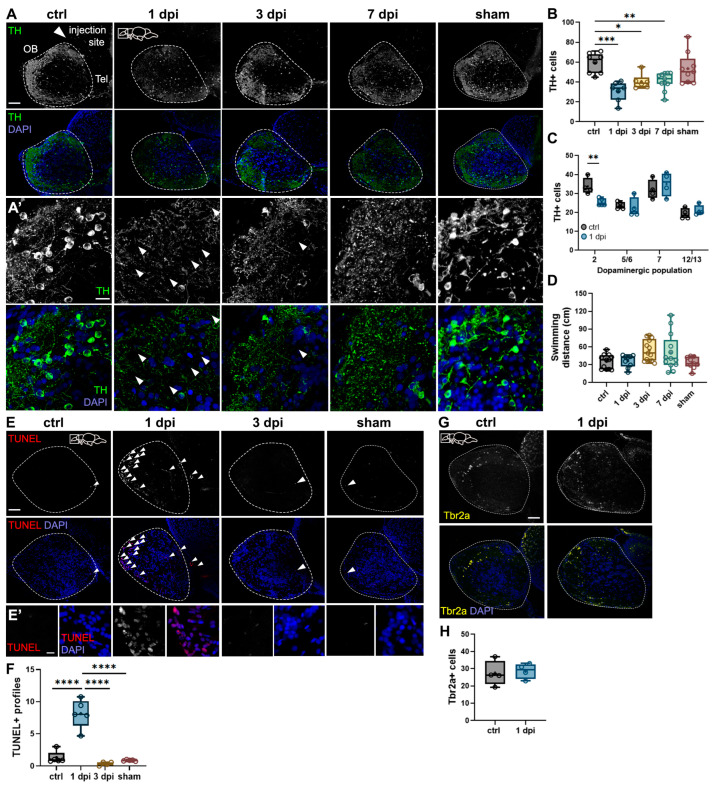
Effects of 6-OHDA injections on the zebrafish brain. (**A**,**A′**) Tyrosine hydroxylase (TH) immunohistochemistry of sagittal sections of the OB from control, 1-day post-injection (dpi), 3 dpi, 7 dpi, and sham fish. 6-OHDA injection site at the dorsal telencephalic ventricle near the telencephalon (Tel) is shown with a white arrowhead in the top left panel. Dotted lines indicate the OB. (**A′**) Magnified views of (**A**). Increased spacing in TH+ staining is indicated with white arrowheads. Green: TH; blue: DAPI. Scale bars: 100 µm in (**A**); 20 µm in (**A′**). (**B**) Quantification of the average number of TH+ cells in sagittal sections of the OB from (**A**) (*n* = 6–11). ANOVA: F (4, 36) = 8.34, *p* < 0.0001. ctrl vs. 1 dpi *p* = 0.0001, ctrl vs. 3 dpi *p* = 0.0025, ctrl vs. 7 dpi *p* = 0.0088. (**C**) Quantification of the average number of TH+ cells in dopaminergic populations 2, 5/6, 7, and 12/13 from sagittal sections of control and 1 dpi fish (*n* = 4). For population 2: t = 3.883, df = 7, *p* = 0.0060. (**D**) Quantification of swimming responses in control, 1 dpi, 3 dpi, 7 dpi, and sham fish (*n* = 9–12). (**E**) (TdT) dUTP Nick-End Labeling (TUNEL) staining in sagittal sections of the OB from control, 1 dpi, 3 dpi, and sham groups. TUNEL+ profiles are indicated with white arrowheads. Red: TUNEL; blue: DAPI. Scale bars: 100 µm in (**E**); 20 µm in (**E′**). (**F**) Quantification of average TUNEL+ profiles in OB sections from (**E**) (*n* = 4–5). ANOVA: F (3, 15) = 40.03, *p* < 0.0001. ctrl vs. 1 dpi *p* < 0.0001, 1 dpi vs. 3 dpi *p* < 0.0001, 1 dpi vs. sham *p* < 0.0001. (**G**) Tbr2a immunohistochemistry of sagittal sections of the OB from control and 1 dpi groups. Yellow: Tbr2a; blue: DAPI. Scale bar: 100 µm. (**H**) Quantification of Tbr2a+ cells in OB sections from (**G**) (*n* = 4). Box plots indicate mean (+), quartiles (boxes) and range (whiskers). One-way ANOVA or unpaired *t*-test for (**C**), * *p* < 0.005, ** *p* < 0.001, *** *p* = 0.0009, **** *p* < 0.0001.

We aimed to develop a model of olfactory dysfunction associated with dopaminergic loss without motor impairment. To achieve this, we injected 6-hydroxydopamine (6-OHDA) into the dorsal telencephalic ventricle of adult zebrafish, aiming to target dopaminergic (DA) periglomerular neurons in the olfactory bulb (OB). To assess the impact of 6-OHDA injections in the OB, we examined sagittal brain sections immunostained for tyrosine hydroxylase (TH), a marker of dopaminergic interneurons [23,24]. At 1-day post-injection (dpi), we observed a significant loss of TH+ neuronal somata compared to controls (Figure 1A,B). However, no further decrease was detected at 3 or 7 dpi (Figure 1A,B), suggesting that 6-OHDA-induced DA neuron loss peaks at 1 dpi, consistent with previous reports [22]. To confirm that this DA loss is due to 6-OHDA specifically and not to the injection itself, we used a sham (i.e., vehicle) injection in a group of fish and found no changes in the number of TH neurons in the OB (Figure 1A,B).

High-magnification images revealed a notable disruption of DA neuron dendritic arbors in all 6-OHDA-injected groups compared to controls (Figure 1A′). In the 1 dpi group, TH+ puncta were more widely spaced across the parenchyma (arrowheads), indicating dendritic disorganization. However, by 7 dpi, the dendritic structure appeared closer to control levels than to 1 dpi (Figure 1A′), suggesting that the remaining periglomerular neurons in the OB exhibit dendritic plasticity in response to injury.

Next, we assessed whether the 6-OHDA injection affected other dopaminergic nuclei in the brain by quantifying TH+ profiles in dopaminergic populations 2 (subpallium), 7 (preoptic nucleus), 5/6, and 12/13 (posterior tuberculum) (we used the nomenclature reported in [22]). We focused on population 2 due to its proximity to the site of injection, and populations 5/6, 7, and 12/13 since these mediate motor function [24,25,26]. We found that 6-OHDA significantly reduced the number of dopaminergic neurons in the subpallium but not in other nuclei (Figure 1C). Then, we assessed potential motor impairments due to the 6-OHDA injection. We quantified swimming distances in control, injected, and sham fish in an open arena. No significant differences were found across groups (Figure 1D), indicating that neither 6-OHDA nor vehicle i.c.v. injections impair swimming performance, consistent with the results that motor nuclei remained unaffected with the 6-OHDA injection.

To determine whether 6-OHDA induces apoptosis, we performed a terminal deoxynucleotidyl transferase (TdT) dUTP Nick-End Labeling (TUNEL) assay. At 1 dpi, we observed a significant increase in TUNEL+ profiles in the OB glomerular layer, where periglomerular neurons reside (Figure 1E,E′,F). By 3 dpi, TUNEL+ cell numbers returned to control levels (Figure 1E,F), indicating that apoptosis occurs rapidly and is not sustained [27]. This aligns with our finding that TH+ cell loss does not persist beyond 1 dpi (Figure 1B). Further confirming that DA loss is due to 6-OHDA and not to the injection itself, sham injections did not cause apoptosis in the OB (Figure 1E,F).

An additional analysis revealed that 6-OHDA injections selectively target dopaminergic but not glutamatergic bulbar neurons. We used Tbr2a to label a subset of mitral cells [28,29] and found no difference in 6-OHDA-injected animals compared to controls (Figure 1G,H). Collectively, these results demonstrate that 6-OHDA injections in the dorsal telencephalic ventricle selectively target DA periglomerular neurons in the OB without affecting posterior dopaminergic nuclei and impairing motor function.

### 2.2. Periglomerular Cell Loss Is Associated with Severe Synaptic Disruption in the OB

**Figure 2:**
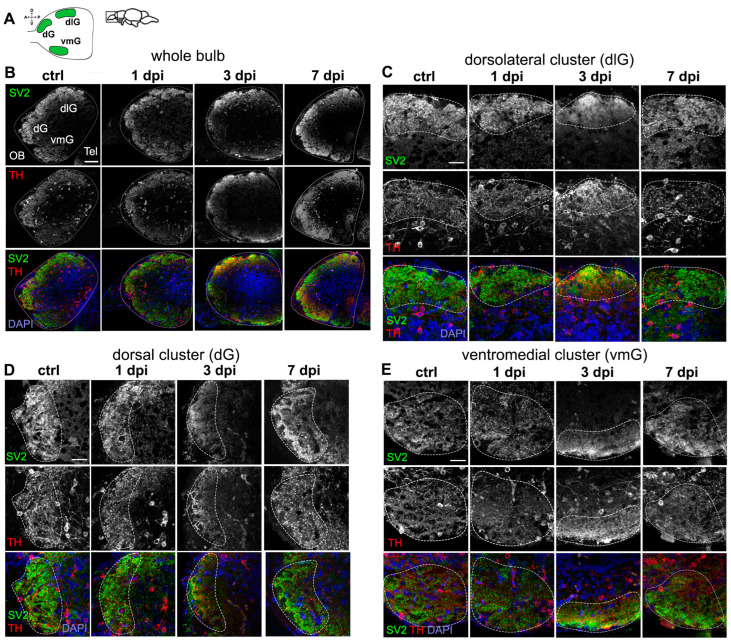
Dysregulation of synaptic contacts in the OB caused by 6-OHDA. (**A**) Schematic diagram of a sagittal view of the OB, indicating three glomerular clusters: dorsal (dG), dorsolateral (dlG), and ventromedial (vmG). (**B**–**E**) Double immunohistochemistry of synaptic vesicle protein 2 (SV2) and tyrosine hydroxylase (TH) in sagittal sections of the OB from controls, 1 dpi, 3 dpi, and 7 dpi groups. Representative images taken from 5–7 animals analyzed per group. Dotted lines indicate the glomerular clusters selected. Whole bulb (**B**), and magnified views of: (**C**) dlG cluster, (**D**) dG cluster, and (**E**) vmG cluster. Green: Tbr2a; red: TH; blue: DAPI. Scale bars: 100 µm in (**B**); 50 µm in (**C**–**E**).

Olfactory sensory neurons (OSNs) form the first synapses of the olfactory pathway within spherical structures in OB parenchyma called glomeruli. These synapses, essential for odor processing, consist of OSN presynaptic termini and the dendrites of glutamatergic neurons (i.e., mitral and tufted cells [30]) and dopaminergic (DA) interneurons (i.e., periglomerular cells [9]). Since 6-OHDA targets periglomerular cells and disrupts their dendritic arbors (Figure 1), we examined its effects on olfactory synaptic contacts. To do this, we qualitatively analyzed immunostained sections against the presynaptic marker synaptic vesicle protein 2 (SV2), which labels olfactory axon terminals, along with TH to visualize DA neurons. We focused on three large glomerular clusters apparent in sagittal OB sections: dorsal (dG), dorsolateral (dlG), and ventromedial (vmG) clusters (Figure 2A,B).

Individual glomeruli were identified based on SV2 staining (dotted lines in Figure 2C–E). We observed a pattern of synaptic disorganization followed by recovery across all glomeruli, indicating the widespread impact of 6-OHDA injections throughout the OB. In control fish, glomerular clusters appeared as distinct, round SV2-stained structures containing individual, smaller glomeruli [31]. TH-stained dendrites were present within these spaces—where they form synaptic contacts—while DA somata were located outside the cluster (Figure 2B,C).

At 1 dpi, TH+ puncta and somata showed clear disorganization, yet individual SV2-stained glomeruli remained distinguishable, albeit smaller than in controls (Figure 2B,C). By 3 dpi, glomerular organization was nearly lost, with no distinct glomeruli visible (Figure 2B,C). Both SV2 and TH staining were relocalized closer to the outer layers of the OB, suggesting synaptic decoupling and degeneration. By 7 dpi, most glomerular structures had recovered, resembling those of control fish (Figure 2B,C). These findings suggest that while DA neuron loss peaks at 1 dpi, olfactory axon disorganization and synaptic degeneration occur more gradually over the following days. By 7 dpi, both periglomerular cell dendrites and olfactory presynaptic terminals appear remodeled and reorganized, highlighting the high plasticity of neurons within the olfactory system.

### 2.3. Periglomerular and Synaptic Degeneration in the OB Lead to Impaired Olfactory Responses to Selective Odorants Without Altering Locomotion

**Figure 3:**
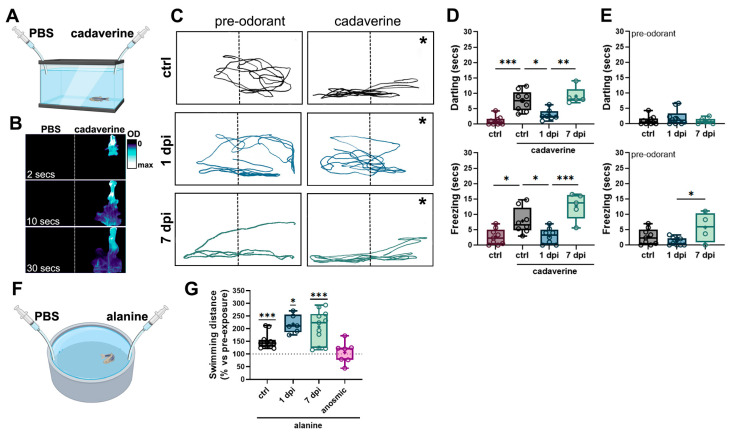
6-OHDA injections selectively reduce olfactory-mediated responses. (**A**) Schematic representation of the behavioral chamber used for studying responses to cadaverine. A rectangular, narrow chamber with a camera located in front was used to study fish’ vertical displacement following cadaverine exposure. (**B**) Time course of a dye distribution in the behavioral chamber from (**A**) indicating that odorant solutions remain in half of the chamber (indicated by a dotted line) during 30 s after cadaverine delivery. In (**C**), the asterisk (*) indicates the position where cadaverine solution was administered. (**C**) Representative swimming trajectories of zebrafish pre-(30 s) and post-(30 s) cadaverine delivery in controls (upper panels), 1 dpi (middle panels), and 7 dpi (lower panels) fish. (**D**) Quantification of swimming responses to cadaverine in controls, 1 dpi, 3 dpi, and 7 dpi (*n* = 5–10 fish). Top panel: time spent darting after cadaverine exposure. ANOVA F (3, 27) = 19.93, *p* < 0.0001. ctrl vs. control cad *p* < 0.0001, ctrl cad vs. 1 dpi *p* = 0.0033, 1 dpi vs. 7 dpi *p* = 0.0007. Bottom panel: time spent freezing after cadaverine exposure. ANOVA F (3, 26) = 12.28, *p* < 0.0001. ctrl vs. control + cad *p* = 0.0206, ctrl cad vs. 1 dpi *p* = 0.0379, 1 dpi vs. 7 dpi *p* = 0.0002. (**E**) Quantification of darting (top panel) and freezing (bottom panel) before odorant exposure (*n* = 4–9 fish). For freezing: ANOVA F (2, 19) = 4.088, *p* = 0.0334. 1 dpi vs. 7 dpi *p* = 0.0261. (**F**) Schematic representation of the behavioral chamber used for studying responses to alanine. A larger circular chamber with an overhead camera was used to study fish’ swimming distance following alanine exposure. (**G**) Quantification of swimming responses to alanine compared against pre-odorant exposure in controls, 1 dpi, 3 dpi, 7 dpi, and anosmic fish (*n* = 6–12 fish). One-sample Wilcoxon test when compared to baseline of 100% (no change). ctrl = *p* = 0.0005, 1 dpi *p* = 0.0312, 7 dpi *p* = 0.0010. * *p* < 0.005, ** *p* < 0.001, *** *p* = 0.0009.

Having established that 6-OHDA injections do not impair motor function, we sought to assess whether DA loss leads to olfactory deficits by employing olfactory-mediated behavioral assays. For this, we used two behavioral arenas to record responses to different odorants (Figure 3A,F). In the first experiment, we used a narrow rectangular tank to evaluate aversive responses to cadaverine, including freezing and darting (Figure 3A).

Individual fish were recorded for 30 s before (pre-odorant) and after cadaverine (cad) exposure. The odorant remained confined to one half of the chamber (Figure 3B). Control fish exhibited typical olfactory-mediated avoidance behaviors, such as increase in darting (Figure 3D, top panel) and freezing (Figure 3D, bottom panel) compared to non-exposed controls. In contrast, 1 dpi fish maintained similar swimming patterns after odorant exposure (Figure 3C, middle panel) and did not exhibit erratic swimming or freezing following exposure to cadaverine (Figure 3D), indicating an impaired olfactory response to this odorant. Since extensive synaptic remodeling was observed in the OB by 7 dpi, we tested whether olfactory function could recover. In line with our predictions, 7 dpi fish exhibited swimming trajectories and behaviors similar to controls (Figure 3C, bottom panel; Figure 3D), significantly different from the 1 dpi group, suggesting that olfactory responses to cadaverine are restored by 7 dpi. To determine whether 6-OHDA altered baseline darting and freezing behaviors, we analyzed pre-odorant trial data. Ultimately, 1 dpi fish showed no differences in darting or freezing compared to controls (Figure 3E). However, in the 7 dpi group, freezing time was significantly increased compared to the 1 dpi group, but not to controls (Figure 3E).

To assess whether olfactory dysfunction was more widespread, we assessed olfactory responses to alanine, an amino acid that elicits attractive and appetitive behaviors, reflected as increased swimming distance and speed [32] (Figure 3F). As expected, we found that control fish responded to alanine by significantly increasing the swimming distance after alanine exposure, compared to a 100% baseline (vs. non-exposed fish, Figure 3G). Both 1 dpi and 7 dpi fish exhibited similar responses to alanine, suggesting that olfactory responses to this odorant remained intact (Figure 3G). Since alanine is palatable for some teleost species [33], we investigated whether the observed behavioral responses could be attributed to gustation. For this, we generated a group of anosmic fish by occluding the nares with surgical adhesive. These fish did not increase their swimming distance following alanine exposure (Figure 3G), confirming that the responses to alanine observed were mediated by olfaction.

Overall, our results demonstrate that 6-OHDA injections selectively disrupt olfactory responses. While fish lose the ability to detect cadaverine, their response to alanine remains intact. Notably, cadaverine sensitivity is restored by 7 dpi. Together, these findings confirm that our model successfully replicates prodromal PD with olfactory dysfunction in the absence of motor impairment.

### 2.4. Astroglial Cells Are Activated in the OB Following 6-OHDA Injections

Neuroinflammation is a hallmark of Parkinson’s disease (PD) pathology, with sustained astroglial and microglial activation contributing to synaptic and neuronal degeneration [34,35]. In contrast, zebrafish exhibit a rapid resolution of post-injury neuroinflammatory responses without forming a permanent astroglial scar [36], which has been shown to hinder repair in mammalian brains [37]. Thus, we sought to determine whether 6-OHDA ventricular injections induce astroglial activation in the OB.

**Figure 4:**
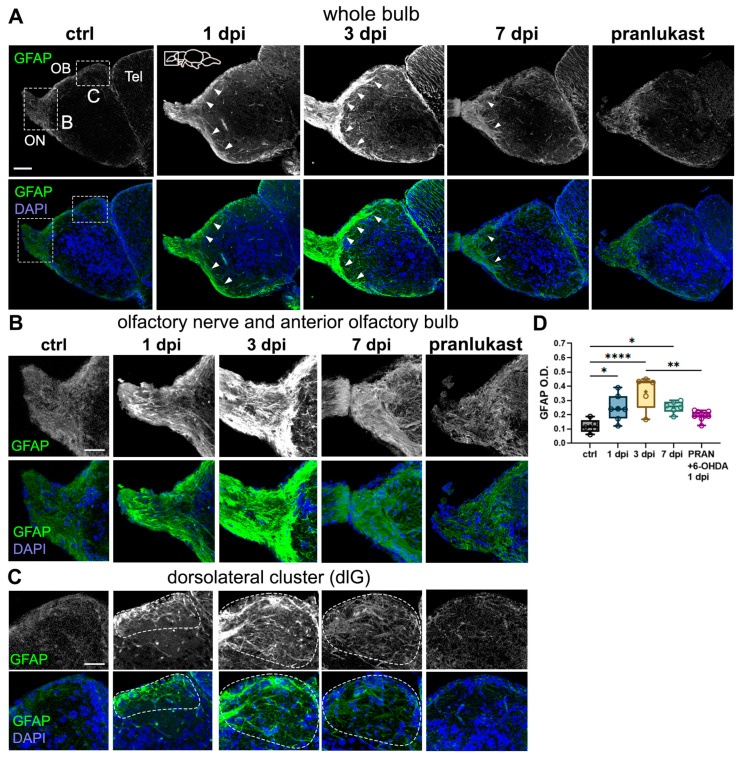
6-OHDA injections lead to astroglial activation in the OB. (**A**–**C**) Glial fibrillary acidic protein (GFAP) immunohistochemistry in sagittal sections of the OB from controls, 1 dpi, 3 dpi, 7 dpi, and 6-OHDA with the anti-inflammatory drug pranlukast (PRAN). Whole bulb (**A**) and magnified views of: (**B**) the olfactory nerve and anterior OB, and (**C**) the dorsolateral (dlG) glomerular cluster (indicated by dotted lines). GFAP+ staining along the olfactory nerve layer (ONL) is indicated with white arrowheads. Green: GFAP; blue: DAPI. Scale bars: 100 µm in (**A**); 50 µm in (**B**,**C**). (**D**) Quantification of GFAP O.D. in OB sections from (**A**) (*n* = 5–9). ANOVA: F (4, 27) = 8.478, *p* = 0.0001. ctrl vs. 1 dpi *p* = 0.0306, ctrl vs. 3 dpi *p* < 0.0001, ctrl vs. 7 dpi *p* = 0.0275. 1 dpi vs. 3 dpi *p* < 0.0001. 3 dpi vs. PRAN *p* = 0.0017. Box plots indicate mean (+), quartiles (boxes) and range (whiskers). One-way ANOVA, * *p* < 0.005, ** *p* < 0.001, **** *p* < 0.0001.

To investigate this, we analyzed OB tissue immunostained against the astroglial marker, glial fibrillary acidic protein (GFAP). Consistent with previous reports, GFAP staining was observed in the olfactory nerve (ON) and the outermost OB layer, the olfactory nerve layer (ONL), both of which are composed of olfactory axons stemming from the OSNs from the olfactory epithelium (OE) (Figure 4A, arrowheads) [38].

In 6-OHDA-injected fish, we detected a significant increase in GFAP expression at all timepoints, peaking at 3 dpi (Figure 4A,B). To determine whether this astroglial activation resulted from 6-OHDA or the injection procedure itself, we co-injected 6-OHDA with the anti-inflammatory drug pranlukast (PRAN) and examined the tissue at 1 dpi. Our findings revealed no significant differences between the control and PRAN-treated groups (Figure 4A,B), indicating that the 6-OHDA-mediated inflammatory response can be attenuated with anti-inflammatory treatment.

Further analysis confirmed a marked increase in GFAP+ staining and branching across the ON and ONL in all injected groups, except for the PRAN-treated group (Figure 4B). We observed a dramatic increase in GFAP+ processes surrounding the dorsolateral glomerulus (dlG, dotted line), particularly at 3 dpi, where astroglial processes distinctly outlined the glomerular cluster. This pattern was absent in the control and PRAN groups (Figure 4C). Interestingly, the peak in astroglial activation at 3 dpi coincides with the timepoint at which the synaptic disorganization is the most pronounced (Figure 2), suggesting a role for astroglia in synaptic remodeling.

### 2.5. 6-OHDA Promotes Microglial Activation and Leukocyte Migration to the OB

A key component of the neuroinflammatory response, which is also heightened in PD, is microglial activation [39]. To further investigate the neuroimmune response to 6-OHDA injections, we performed histological characterization using the leukocyte/microglial marker lymphocyte cytosolic protein 1 (Lcp1, also known as L-plastin). Lcp1 is primarily expressed in microglia within the central nervous system (CNS) and in peripheral leukocytes. Under normal physiological conditions, peripheral immune cells are scarce in the CNS, making Lcp1 a bona fide microglial marker [40].

**Figure 5:**
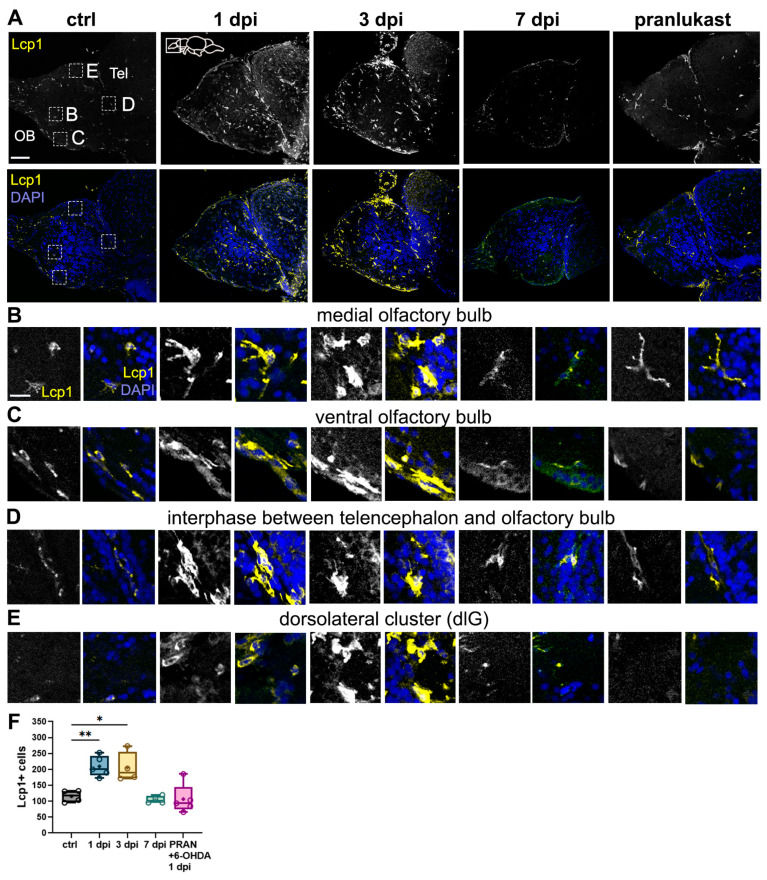
Microglial activation and leukocyte migration in the OB following 6-OHDA injection. (**A**–**E**) Lymphocyte cytosolic protein 1 (Lcp1) immunohistochemistry in sagittal sections of the OB from controls, 1 dpi, 3 dpi, 7 dpi, and 6-OHDA with the anti-inflammatory drug pranlukast (PRAN). Whole bulb (**A**) and magnified views of: (**B**) middle region of the OB, (**C**) dlG cluster region, (**D**) ventral region, and (**E**) the interphase between the telencephalon and the OB. Yellow: Lcp1; blue: DAPI. Scale bars: 100 µm in (**A**); 20 µm in (**B**–**E**). (**F**) Quantification of Lcp1+ cells in OB sections from (**A**) (*n* = 4–5). ANOVA: F (4, 17) = 10.66, *p* = 0.0002. ctrl vs. 1 dpi *p* = 0.0074, ctrl vs. 3 dpi *p* = 0.0151, ctrl vs. 7 dpi *p* = 0.0028, 1 dpi vs. 7 dpi *p* = 0.0028, 1 dpi vs. PRAN *p* = 0.0019, 3 dpi vs. 7 dpi *p* = 0.0061, 3 dpi vs. PRAN *p* = 0.0046. Box plots indicate mean (+), quartiles (boxes) and range (whiskers). One-way ANOVA, * *p* < 0.005, ** *p* < 0.001.

In control fish, we observed sparse Lcp1+ microglial cells distributed across the OB, with most cells displaying a stellate morphology characteristic of a resting state (Figure 5A,B) [41]. However, in 1 dpi and 3 dpi fish, there was a significant increase in Lcp1+ cells in the OB, suggesting an infiltration of peripheral leukocytes (Figure 5A,F). Higher magnification images of the OB periphery (Figure 5C) and of the interphase between the telencephalon and OB (Figure 5D) confirmed a noticeable increase in Lcp1+ cells at these timepoints, indicative of cell infiltration. Consistent with our earlier findings of astroglial activation around glomerular clusters, particularly in the dorsolateral glomerulus (dlG) (Figure 4C), we also observed an accumulation of Lcp1+ cells in this region at 1 dpi and 3 dpi (Figure 5E). In addition to increased cell migration, we noted striking changes in the size and morphology of Lcp1+ cells in the 1 dpi and 3 dpi groups, indicating activation states. These alterations, including larger somata with extensive processes (indicative of migration) and rounded, enlarged somata (suggestive of phagocytic activity), were most pronounced at 3 dpi (Figure 5B–E). Interestingly, by 7 dpi, the leukocytic/microglial response had largely subsided (Figure 5A,B), and this response was notably hampered in fish co-treated with PRAN (Figure 5A,B). Taken together, these findings demonstrate that 6-OHDA injections trigger robust microglial and leukocytic activation and migration in the OB, a response that resolves by 7 dpi. Notably, the peak activation and phagocytic activity of Lcp1+ cells at 3 dpi align with the observed increase in astrocytic activity at this same timepoint (Figure 4), suggesting that astroglial and microglial cells work collaboratively at axonal and synaptic sites to facilitate their remodeling and recovery.

### 2.6. 6-OHDA-Mediated Changes in the OB Cause Retrograde Degeneration and Cell Proliferation in the OE

**Figure 6:**
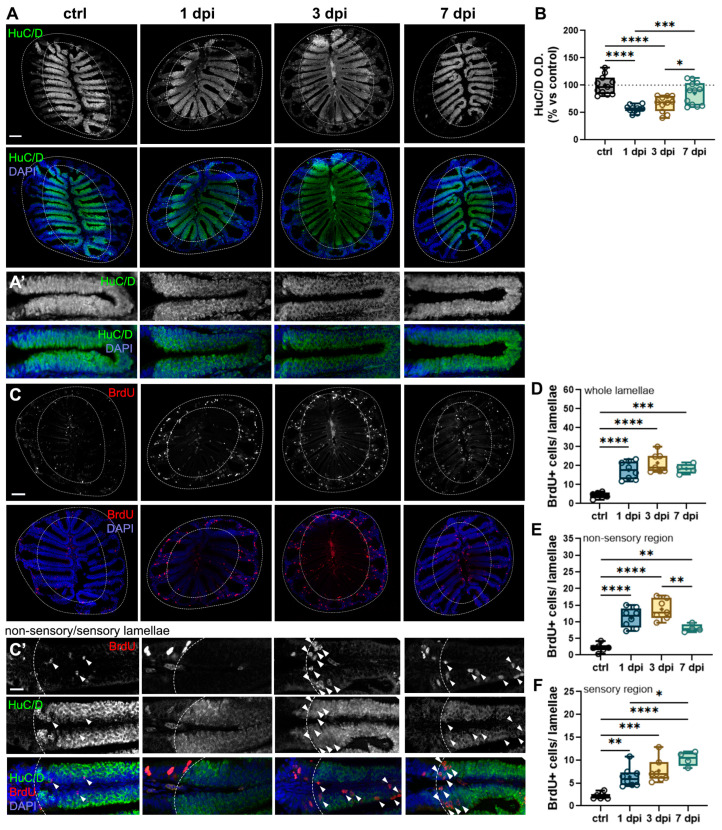
6-OHDA injections in the OB cause retrograde degeneration in the OE. (**A**,**A′**) HuC/D immunohistochemistry of OE sections from controls, 1 dpi, 3 dpi, and 7 dpi groups. (**A’**) are magnified views of the OSN-containing sensory lamellae (HuC/D+) from (**A**). Scale bars: 100 µm in (**A**); 20 µm in (**A′**). (**B**) Quantification of percent change (vs. control) in optical density (O.D.) of HuC/D in the OE from (**A**) (*n* = 11–12). ANOVA F (3, 41) = 16.78, *p* < 0.0001. ctrl vs. 1 dpi *p* < 0.0001, ctrl vs. 3 dpi *p* < 0.0001, 1 dpi vs. 7 dpi *p* = 0.0005, 3 dpi vs. 7 dpi *p* = 0.0286. (**C**,**C′**) Double immunohistochemistry of BrdU and HuC/D in sections of the OE of controls, 1 dpi, 3 dpi, and 7 dpi fish. (**C′**) Magnified views of (**C**), showing the sensory and non-sensory regions of the OE lamellae (indicated by dashed lines and HuC/D staining). BrdU+ cells found in the sensory area (HuC/D+) are indicated with white arrowheads. Red: BrdU; green: HuC/D; blue: DAPI. Scale bars: 100 µm in (**C**); 20 µm in (**C′**). (**D**) Quantification of BrdU+ cells in whole lamellae from (**A**) (*n* = 4–8). ANOVA F (3, 21) = 21.26, *p* < 0.0001. ctrl vs. 1 dpi *p* < 0.0001, ctrl vs. 3 dpi *p* < 0.0001, ctrl vs. 7 dpi *p* = 0.0002. (**E**) Quantification of BrdU+ cells in the non-sensory lamellae from (**A**) (*n* = 4–8). ANOVA F (3, 21) = 25.83, *p* < 0.0001. ctrl vs. 1 dpi *p* < 0.0001, ctrl vs. 3 dpi *p* < 0.0001, ctrl vs. 7 dpi *p* = 0.0073, 3 dpi vs. 7 dpi *p* = 0.0075. (**F**) Quantification of BrdU+ cells in the sensory lamellae (HuC/D+) from (**A**) (*n* = 4–8). ANOVA F (3, 21) = 14.86, *p* < 0.0001. ctrl vs. 1 dpi *p* = 0.0046, ctrl vs. 3 dpi *p* = 0.0004, ctrl vs. 7 dpi *p* < 0.0001, 3 dpi vs. 7 dpi *p* = 0.0075, 1 dpi vs. 7 dpi *p* = 0.0205. Box plots indicate mean (+), quartiles (boxes) and range (whiskers). One-way ANOVA, * *p* < 0.005, ** *p* < 0.001, *** *p* = 0.0009, **** *p* < 0.0001.

Given the bidirectional communication between the peripheral olfactory epithelium (OE) and the olfactory bulb (OB), we aimed to determine whether the neuronal and synaptic alterations induced by 6-OHDA affected the OE through retrograde degeneration mechanisms [42,43]. To assess this, we analyzed sections of the olfactory organ immunostained for HuC/D, a pan-neuronal marker in zebrafish. The olfactory organ comprises both sensory (HuC/D+) and non-sensory epithelium, (delineated in dotted lines, Figure 6A). We observed a significant reduction in HuC/D staining in the 1 dpi and 3 dpi groups compared to controls (Figure 6A,A′,B), suggesting olfactory sensory neuron (OSN) loss or dysfunction in the OE. However, there were no noticeable changes in the overall size or gross morphology of the olfactory organ (Figure 6A). By 7 dpi, HuC/D staining had returned to control levels, indicating OSN recovery (Figure 6A,A′,B).

The OE is known for its rapid regenerative capacity following various types of damage [43]. Given that OSNs recovered by 7 dpi, we hypothesized that neurogenesis was upregulated following the injection, thus facilitating OE repair. To test this, we labeled newborn cells generated within the first 24 h after 6-OHDA injection with bromodeoxyuridine (BrdU) and tracked them at different timepoints. Our results revealed a significant increase in BrdU+ profiles across the entire lamellae in all injected groups compared to controls (Figure 6C,D).

Additionally, the OE contains two main progenitor populations: globose basal cells (GBCs), which reside in the lateral, non-sensory regions of the OE and are constitutively proliferative; and horizontal basal cells (HBCs), located along the basal lamina of the sensory epithelium (Figure 6C′, delineated by dotted lines). Under normal conditions, HBCs remain quiescent but become activated in response to epithelial damage [44]. By analyzing the localization of BrdU+ cells within the epithelium, we assessed neurogenesis, as neurons derived from progenitors migrate toward the sensory lamellae, typically positioned more apically than HBCs, which remain near the basal lamina.

Our findings showed an increase in BrdU+ cells in both the non-sensory and sensory regions in all injected groups compared to controls, suggesting activation of both types of progenitors (Figure 6C–F). Notably, the number of BrdU+ cells in the non-sensory epithelium exhibited a decreasing trend in the 7 dpi group, as indicated by a significant difference compared to the 1 dpi group (Figure 6E). Conversely, in the sensory epithelium, we observed the opposite trend: while all injected groups displayed a significant increase in BrdU+ cells compared to controls (Figure 6C′, arrowheads; F), BrdU+ profiles were further elevated at 7 dpi, as indicated by a significant increase relative to the 1 dpi group (Figure 6F). Together, these results indicate that 6-OHDA injections lead to retrograde degeneration of OSNs in the OE, followed by recovery by 7 dpi. This process is accompanied by increased cell proliferation and neurogenesis in the OE, particularly evident at 7 dpi—coinciding with the restoration of olfactory axonal synaptic morphology and olfactory function (Figure 2 and Figure 3).

### 2.7. Peripheral Leukocytes Migrate to OE’s Sensory Epithelium Following 6-OHDA Injections

**Figure 7:**
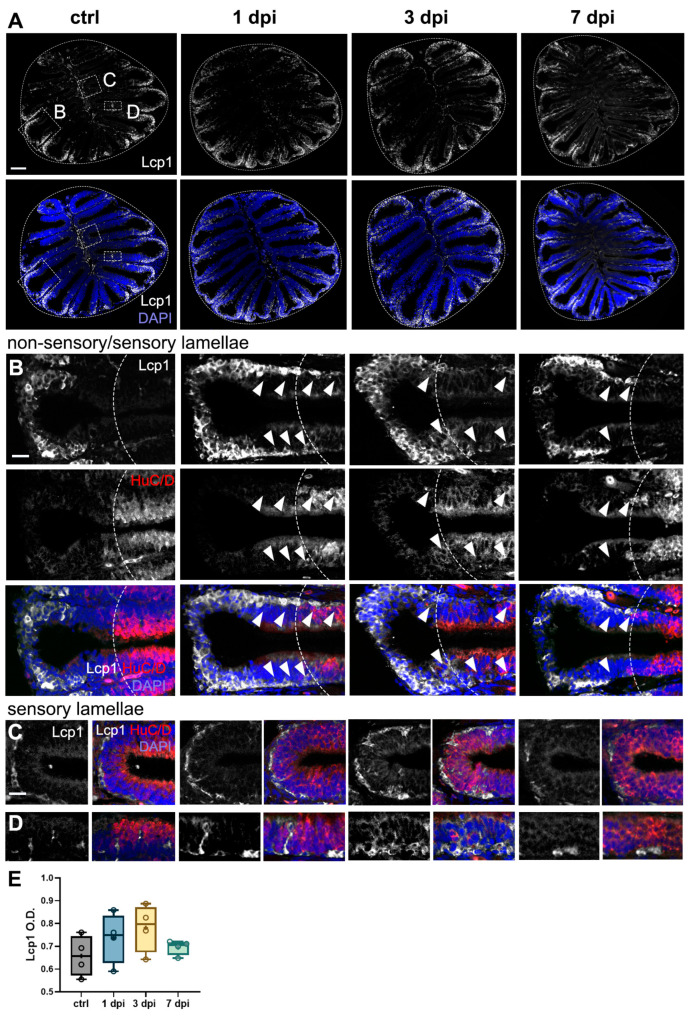
Leukocytic migration within the OE caused by 6-OHDA injections in the OB. (**A**–**D**) Lymphocyte cytosolic protein 1 (Lcp1) immunohistochemistry in sections of the OE from controls, 1 dpi, 3 dpi, and 7 dpi. Whole OE (**A**) and magnified views of the following: (**B**) distal epithelial fold region, where the OSN-containing sensory region is indicated by HuC/D staining and a dotted line; (**C**) medial sensory region (HuC/D+) adjacent to the central raphe; and (**D**) sensory epithelium (HuC/D+). Migration of Lcp1+ cells is indicated by white arrowheads. White: Lcp1; blue: DAPI. Scale bars: 100 µm in (**A**); 20 µm in (**B**–**D**). (**E**) Quantification of Lcp1+ cells in OE sections from (**A**) (*n* = 4). ANOVA: F (3, 12) = 1.42, *p* = 0.2840. Box plots indicate mean (+), quartiles (boxes), and range (whiskers).

We hypothesized that the observed alterations in the OE would be accompanied by an increase in inflammatory processes. To investigate this, we performed Lcp1 staining in OE sections to assess the presence of peripheral leukocytes in the OE. In control fish, we identified a resident population of Lcp1+ cells primarily localized within the non-sensory lamellae, with only a few individual cells dispersed throughout the sensory epithelium (Figure 7A), consistent with previous reports describing a similar organization of neutrophils (the most abundant leukocyte) in the OE [45].

Interestingly, we did not detect significant differences in Lcp1+ optical density (O.D.) across groups (Figure 7A,E). We assessed O.D. as the high density of Lcp1+ cells in the OE precluded reliable individual cell counts. Upon closer examination of magnified views of the epithelia at the interphase between the non-sensory and sensory regions (Figure 7B, dotted lines), it was revealed that, in the 1 dpi and 3 dpi groups, Lcp1+ cells migrated toward the HuC/D+ sensory region (Figure 7B, arrowheads). By 7 dpi, the distribution of leukocytes resembled that of control fish, with most cells residing in the non-sensory epithelium (Figure 7B). We also examined the medial epithelial folds adjacent to the central raphe, a known site of immune cell infiltration into the OE [45,46]. At 1 dpi and 3 dpi, we observed an increased presence of Lcp1+ cells lining the basal end of the epithelium near the raphe, suggestive of cell migration, but this increase was not evident at 7 dpi (Figure 7C). Additionally, in the 1 dpi and 3 dpi groups, leukocytes were embedded throughout the width of the sensory epithelium, suggesting that these immune cells migrate both horizontally and apically within the sensory lamellae (Figure 7D). Collectively, these findings indicate that 6-OHDA injections in the OE lead to the redistribution of resident Lcp1+ leukocytes, shifting from the non-sensory lamellae to the OSN-rich sensory regions.

## 3. Discussion

In this study, we developed a novel model of olfactory impairment related to dopaminergic loss, one of the features of prodromal Parkinson’s disease (PD). To this end, we injected 6-OHDA into the dorsal telencephalic ventricle to target dopaminergic (DA) periglomerular neurons in the olfactory bulb (OB) in adult zebrafish. Importantly, in this model, fish swimming performance is not impaired, which enabled us to study the effects of dopaminergic loss on olfactory function by using behavioral assays. Importantly, by using zebrafish, we were able to explore neuroplasticity and regenerative processes that are absent in mammalian models of dopaminergic loss and PD.

While this novel model proves a useful tool, it comes with some caveats. For example, it does not replicate the progressive and multifactorial nature of the disease, including the OB atrophy observed in human patients and the α-synuclein pathology that is not possible to achieve with 6-OHDA injections.

### 3.1. 6-OHDA-Mediated Dopaminergic Loss in the OB Leads to Olfactory Dysfunction

Our findings reveal that 6-OHDA injections led to a decreased olfactory-mediated response to cadaverine, an odorant that elicits avoidance behaviors, while responses to alanine, an odorant prompting attraction and appetitive behaviors, remained unchanged. This selective olfactory impairment suggests that certain olfactory circuits may be more vulnerable to dopaminergic loss than others, possibly reflecting differential susceptibility among olfactory sensory neuron (OSN) subtypes. Given that amino acids serve as cues for detecting food sources, the teleost olfactory system has adapted to prioritize amino acid detection, with a greater proportion of OSNs dedicated to processing these stimuli compared to aversive odorants [47,48]. Cadaverine is recognized by a subset of ciliated OSNs that extend projections to a single glomerulus in the dorsolateral cluster (dlG) [48,49], while amino acids, such as alanine, activate a larger group of microvillous OSN that project to lateral glomeruli [50,51].

Importantly, our results confirm prior findings by Godoy et al., who reported that DA neuron ablation in the OB leads to impaired responses to cadaverine [52]. Although there are important differences in the onset of olfactory dysfunction, in both studies, this dysfunction correlates with the peak loss of periglomerular neurons. Godoy et al. employed an elegant chemogenic strategy to ablate DA neurons selectively, which led to peak DA neuron loss and olfactory dysfunction at 7 days post-ablation. We observed olfactory dysfunction as early as 1 day after 6-OHDA exposure, coinciding with the peak DA neuron loss in our model. This discrepancy may be due to the neurotoxic effects of 6-OHDA, which ablates DA neurons more rapidly.

Dopaminergic neuron regeneration in the OB is well documented in zebrafish and other species [22,52,53,54] and is thought to contribute to the recovery of olfactory function following DA loss [21,52]. Our data show that DA neuron numbers at 7 dpi remain unchanged from 1 dpi, indicating that no new neurons have yet been incorporated into the OB. This aligns with previous reports indicating that bulbar neuron regeneration occurs at least two weeks after neuronal birth [22].

Thus, an important contribution of this work is that olfactory recovery occurred independently of periglomerular neuron regeneration, instead coinciding with compensatory synaptic remodeling in the OB, suggesting that plasticity mechanisms contribute to functional restoration before newly generated DA neurons integrate into OB circuits. Future studies should assess whether DA neuron numbers eventually recover in this model of neurotoxicity and, if so, the precise timeline of neuronal replacement.

### 3.2. 6-OHDA Injections in the Dorsal Telenchpehalic Ventricle Selectively Target Dopaminergic Neurons in the OB and Subpallium Without Affecting Motor Function

Here, we show that dopaminergic neurons in the OB and subpallium are susceptible to 6-OHDA injections in the dorsal telencephalic ventricle, while neurons in the preoptic nucleus and posterior tuberculum remain unaffected. Interestingly, Caldwell et al. also reported differential susceptibility of DA populations to 6-OHDA injections in the third ventricle below the optic tectum [22]. They found DA neuron loss in the posterior tuberculum while nuclei in the preoptic area, OB, and subpallium were spared. The reasons for this differential susceptibility in the zebrafish brain are intriguing and remain to be investigated.

In zebrafish, motor behavior is modulated by dopaminergic populations in the posterior tuberculum and the preoptic nucleus [24,25,26], nuclei that remained unchanged in our injection paradigm. These results coupled with normal swimming performance validate our model in studying the effects of dopaminergic loss in the OB without motor impairment. Dopaminergic populations in the subpallium have not been reported to directly modulate olfaction, so the effects of their ablation can be investigated in future studies.

Our findings indicate that 6-OHDA injections selectively target dopaminergic neurons while sparing glutamatergic cells in the OB, suggesting that the olfactory dysfunction observed can be attributed to periglomerular cell loss without affecting mitral cell numbers. Moreover, these results add to our previous report that excitotoxic damage in the zebrafish OB leads to glutamatergic, but not dopaminergic loss [43]. Collectively, these results echo several studies in mammalian models that demonstrate the differential susceptibility of dopaminergic and glutamatergic neurons to different classes of neurotoxins [28].

### 3.3. Neuroinflammatory Processes Parallel Synaptic Disruption and Recovery in the OB

Our results reveal a time-dependent disruption and recovery of synaptic architecture in the OB that mirrors neuroinflammatory responses. Although periglomerular neuron loss peaks at 1 dpi, synaptic dysregulation is most pronounced at 3 dpi, coinciding with increased astroglial and microglial activation. This aligns with previous findings that demonstrate that neuroinflammatory responses in the zebrafish brain play a critical role in repair following neural injury [55,56].

Unlike mammals, zebrafish lack stereotypical stellate GFAP+ astrocytes [57]. In lieu of these astrocytes, GFAP+ cells have similar morphologies and properties as radial glia in regions like the telencephalon and the optic tectum [58,59]. In the olfactory system, GFAP+ astroglial cells have been described as olfactory ensheathing cells (OECs), which envelop olfactory axons from the OE basal lamina to the OB glomerular layer, where axonal termini are located [38]. OECs are unique glial cells that share properties with astrocytes and oligodendrocytes [60,61] as they secrete neurotrophic factors that support axonal survival, growth, and remyelination [62].

A noteworthy feature of the glial response was the enlargement and dynamic nature of GFAP+ processes, which clearly delineated glomerular sites throughout the post-injection period. This suggests that OECs may contribute to synaptic remodeling, possibly through phagocytosis of damaged synapses and/or secretion of neurotrophic factors [61]. Another important role of OECs is to guide immature axons to their OB targets [63]. We demonstrate that 6-OHDA injections stimulate neurogenesis in the OE, likely as a consequence of decreased OSN density. It is possible that activated OECs facilitate proper axonal targeting of newborn OSNs, contributing to the synaptic remodeling observed at 7 dpi.

Furthermore, we observed pronounced microglial activation and leukocyte recruitment in the OB at 1 and 3 dpi, coinciding with peak astroglial responses, supporting the idea of a coordinated neuroimmune response. Under physiological conditions, microglia are the sole resident immune cells in the central nervous system (CNS), where they continuously survey the environment and facilitate homeostasis. In control fish, microglia were sparse and presented a ramified morphology. Following 6-OHDA injections, we report increased numbers of Lcp1+ cells in the OB, indicating that peripheral leukocytes infiltrated the CNS [64]. Accompanying the increase in the number of Lcp1+ cells in the OB, we observed noticeable changes in cell morphology, indicating infiltration and activation [41]. Leukocyte migration was the most evident in the periphery of the OB as well as in the interphase between the telencephalon and the OB, supporting previous reports of these cells infiltrating the brain through blood vessels and ventricles [64]. Infiltrating leukocytes at these locations exhibited a transitioning, motile state, characterized by an enlarged soma with shorter processes.

Activated microglia adopted an ameboid morphology, characterized by large, rounded somata with retracted processes, reflecting a phagocytic state. This morphology closely resembles that of infiltrating macrophages, which aid microglia in debris clearance and secretion of cytokines that modulate the neuroinflammatory response [41,65]. At 3 dpi, we observed an increase in phagocytic Lcp1+ cells at 3 dpi, particularly around the olfactory nerve layer and glomerular clusters, suggesting that active phagocytosis of extracellular and axonal debris contributes to synaptic remodeling. Coupled with our observations of concomitant astroglial responses, we propose that OECs and microglia act synergistically to facilitate recovery and the establishment of new synaptic contacts following periglomerular cell loss. Notably, co-administration of the anti-inflammatory drug pranlukast mitigated both astrocytic and microglial activation, indicating that the inflammatory response mediated by 6-OHDA can be hampered by anti-inflammatory treatment, indicating that OB inflammation is due to 6-OHDA’s neurotoxic insult. Future work will explore whether neuroinflammatory processes are necessary for the synaptic and functional recoveryobserved in our model.

### 3.4. 6-OHDA Injections Lead to Retrograde Degeneration and Neurogenesis in the Peripheral OE

Another major finding of this study is the evidence of retrograde degeneration of OSNs in the OE, indicated by a significant reduction of HuC/D+ OSNs at 1 dpi and 3 dpi, followed by recovery of neuronal density. These results are consistent with prior reports that neurodegeneration in the OB or in the ON lead to retrograde degeneration [28,42,66], underscoring the bidirectional communication between the peripheral and central components of the olfactory system. Our findings suggest that early olfactory deficits in our model likely result from both central and peripheral neuronal degeneration.

OSN dysfunction triggered local cell proliferation and neurogenesis, as indicated by BrdU labeling. Notably, the increase in BrdU+ profiles in the sensory region of the OE lamellae, coincides with the recovery of cadaverine-induced behavioral responses at 7 dpi. Since DA neurons in the OB had not recovered by this timepoint, we propose that recovery of OSN integrity coupled with synaptic remodeling in glomerular sites contribute to olfactory function recovery. Furthermore, the redistribution of leukocytes to the sensory region of the OE following 6-OHDA injections suggest that peripheral immune cells contribute to OSN repair and regeneration, echoing the response of their counterparts in the OB.

## 4. Materials and Methods

### 4.1. Animals

Adult wild-type zebrafish (*Danio rerio*) of both sexes were kept and bred in a filtered aquarium system (Aquaneering, San Diego, CA, USA) located at Hope College’s zebrafish facility. Fish used were 6–12 months old (between 3 and 3.5 cm long). The aquarium room was kept on a 12-h light: 12 h dark cycle at 28 °C. Fish were fed 3 times a day ad libitum, with commercial flake food (Aquaneering, San Diego, CA, USA) and freshly hatched brine shrimp (Brine Shrimp Direct, Ogden, UT, USA) twice and once a day, respectively.

### 4.2. Intracerebroventricular (i.c.v.) 6-OHDA Injections

To ablate dopaminergic neurons in the olfactory bulb, we performed intracerebroventricular (i.c.v.) injections of 6-hydroxydopamine (6-OHDA; Sigma-Aldrich, St. Louis, MO, USA) in the dorsal telencephalic ventricle, at the junction of the olfactory bulb with the telencephalon. For this, fish were anesthetized by submersion in a cooled 0.03% MS222 (tricaine) solution (Sigma-Aldrich, St. Louis, MO, USA) until the fish were unresponsive to a tail pinch. We used a beveled syringe with a 26-gauge needle (Hamilton, Reno, NV, USA) inserted diagonally through the skull into the telencephalic ventricle to inject 0.5 µL of a 10 mM of a 6-OHDA solution (Evans blue 1% *w*/*v* in PBS evans blue; Sigma-Aldrich, St. Louis, MO, USA) at a rate of 0.01 µL/s using an Aladdin syringe pump (World Precision Instruments, Worcester, MA, USA). We determined injection success by observing the diffusion of the injected Evans blue solution within the ventricular system. Fish were left to recover for either 1, 3, or 7 days post-injection (dpi). Fish from the pranlukast (PRAN; Sigma-Aldrich, St. Louis, MO, USA) treatment received an injection of 0.5 µL of a solution containing 10 mM 6-OHDA with 10 mg/mL pranlukast in Evans blue 1% *w*/*v* in PBS. sham fish were injected with 1% Evans blue in PBS. PRAN and sham fish were sacrificed at 1 dpi. Recovered fish were euthanized by over-anesthetization with tricaine, decapitated, and fixed with 4% paraformaldehyde (PFA; Sigma-Aldrich, St. Louis, MO, USA) in PBS for 24 h at 4 °C. The next day, we carefully dissected brains and olfactory organs and prepared the tissue for immunohistochemistry.

### 4.3. Tissue Processing for Immunohistochemistry

After fixation, we incubated tissue in solutions of increasing ethanol concentrations to dehydrate it, followed by an incubation in xylene (Sigma-Aldrich, St. Louis, MO, USA). Dehydrated tissue was embedded in paraffin (Paraplast plus, McCormick Scientific, Berkeley, CA, USA) and rapidly cooled to solidification. The next day, we obtained semi-serial sagittal 10 μm sections that were then adhered to charged slides (ThermoFisher, Waltham, MA, USA).

### 4.4. Cell Proliferation Assays

To determine cell proliferation, we performed a pulse-and-chase assay with the thymidine analog, 5-bromo-2′-deoxyuridine (BrdU, Sigma Aldrich, St. Louis, MO, USA). We administered 10 μL of a 50 mg/mL BrdU solution in PBS intraperitoneally (i.p.) immediately after the i.c.v. 6-OHDA injection. Fish were then allowed to recover as described above.

### 4.5. Immunohistochemistry

We prepared tissue for immunohistochemistry (IHC) by rehydrating it through descending ethanol solutions followed by antigen retrieval with a 10 mM sodium citrate pH 6.0 solution (Sigma-Aldrich, St. Louis, MO, USA) at 100 °C for 10 min. Next, we blocked slides with a buffer containing 3% normal goat serum (NGS; Vector Laboratories, Burlingame, CA, USA) and 0.4% Triton X-100 (Sigma Aldrich, St. Louis, MO, USA) for at least 1 h at RT or overnight at 4 °C. Next, slides were incubated with primary antibodies (Table 1) overnight at RT, washed, and then incubated with fluorescently labeled secondary antibodies for up to 2 h (Table 1). We added 1 µg/mL of 4′,6-diamidino-2-phenylindole (DAPI; BD Pharmingen, Franklin Lakes, NJ, USA) to the secondary antibody solution as a nuclear counterstain. Next, sections were washed and then coverslipped with PVA-DABCO (Sigma Aldrich, St. Louis, MO, USA). Tissue sections were then examined with a confocal laser-scanning microscope Nikon A1 using NIS-Elements software 6.10.01 (Nikon, Tokyo, Japan).

### 4.6. Densitometry

We utilized Adobe Photoshop (Adobe, Mountain View, CA, USA) to assess the optical density (OD) of stained tissue with HuC/D and Lcp1. We quantified and averaged 3 to 5 tissue sections per animal. We used images taken at 20× magnification and converted to 8-bit gray scale to obtain whole-section mean luminosity values, which were then converted to OD by the following formula: OD = −log (intensity of background/intensity of area of interest).

### 4.7. Cell Quantification

Dopaminergic TH (tyrosine hydroxylase), Lcp1+, BrdU+, Tbr2a+, and TUNEL+ somata were manually quantified. We used 4 to 6 sections of images taken at 20× magnification per animal, and quantified and averaged cells per section.

### 4.8. Olfactory-Mediated Behavioral Assays

We analyzed swimming behavioral responses to two odorants, alanine and cadaverine. We used different behavioral chambers to better study different swimming parameters. In all cases, fish were fasted for 48 h in individual tanks before the experiment and were placed individually in the behavioral chambers. A fish underwent 3 or 4 trials per day, and we averaged the parameters of all trials per fish. The number reported is the number of fish, not of individual trials. A trial was defined as 30 min of general acclimation to the chamber, followed by a 30 min silent acclimation period. After both acclimation periods elapsed, a 30 s baseline recording was taken prior to odorant delivery. Then, an odorant solution and PBS were administered simultaneously on opposite sides of the chamber. Recording continued for 30 s following addition of the compounds and zebrafish behavior was analyzed. After each trial, fish were transferred to another chamber with fresh water and left to acclimate for another trial. Fish underwent 3 or 4 behavioral trials per day. To minimize the effect of the experimenters’ presence, the chambers were surrounded by white panels with perforations for the odorant tubes. The odorant tube was kept the same to control for possible leftover solution in the next trial, but the side of the odorant delivery was randomized for each trial.

For studying behavioral responses to cadaverine, fish were placed in rectangular clear tanks (24.8 cm × 9.7 cm × 15.9 cm) outfitted with two surgical tubes on opposite sides. The chambers were filled with 1.5 L of fresh fish water before each trial. We used syringes attached to the tubes to administer 1 mL of a 100 μM cadaverine solution (Sigma-Aldrich) in PBS and vehicle (PBS) in the opposite tube. We used a digital camera positioned in front of the tank and behind a white panel to minimize the effect of its presence on the fish.

For testing responses to alanine, we used a white cylindrical behavioral apparatus with 30 cm diameter outfitted with two surgical tubes found on opposite sides about 8 cm above the base. This chamber was filled with 2.5 L of fresh fish water before each experiment. We used syringes attached to the tubes to administer 3 mL of a 100 μM alanine solution (Sigma-Aldrich, St. Louis, MO, USA) in PBS and vehicle (PBS) in the opposite tube. We used an overhead digital camera positioned 1 m above to capture swimming behavior [39].

We generated anosmic fish by anesthetizing fish in tricaine as described above. Using a fine paintbrush, we applied cyanoacrylate glue on top of both nasal cavities to temporarily occlude the nares.

### 4.9. Video Analysis

All the swimming behaviors we assessed correspond to well-characterized, stereotypical behaviors described extensively in the literature and by our group [39].

For cadaverine analyses, we coded time (in seconds) spent darting and freezing. Darting was characterized as a sudden increased speed of movement and rapid, sharp directional changes. Freezing was characterized by an absence of movement, often accompanied by sinking to the bottom of the tank. We calculated the percent change in response in pre- and post-trials using the formula: percent change = (pre-trial value − post-trial value)/post-trial value × 100 or the percent time spent in erratic swimming or freezing.

For alanine, we used ToxTrac v024.1.1 [67], an animal tracking software, to analyze swimming behavior recordings after exposure. Analyses were divided into 30 s of pre-odorant delivery and 30 s of post-odorant delivery. Each recording was uploaded to iMovie in order to increase the contrast to allow for better detection on ToxTrac. Swimming distances (mm) were obtained from ToxTrac.

### 4.10. Statistical Analyses

Comparisons between groups were carried out using Analysis of Variance (ANOVA) with Tukey’s post hoc tests or unpaired *t*-tests. *p*-values less than 0.05 were considered significant. Behavioral responses to alanine were analyzed using a one-Sample Wilcoxon test with a baseline of 100% to compare the percentage change in response among groups. All statistical analyses were performed in GraphPad Prism 10 Software (GraphPad, San Diego, CA, USA).

## 5. Conclusions

In conclusion, we describe a novel model of olfactory dysfunction associated with dopaminergic loss that can be associated with early PD pathology in adult zebrafish. We report that olfactory dysfunction and synaptic degeneration were recovered by 7 dpi, highlighting the remarkable and unique regenerative abilities of zebrafish and underscoring a unique advantage of our model. Future studies should explore the molecular mechanisms underlying this recovery, including the role of neurotrophic signaling and immune-mediated repair. Our findings emphasize the use of zebrafish as an amenable and highly neuroplastic model for investigating early PD-related olfactory dysfunction, with the potential for the development of recovery interventions for individuals suffering from olfactory loss.

## Figures and Tables

**Table 1:** Antibodies used in the study.

Antibody	Species	Dilution	Source	Cat. Number
HuC/D	Mouse	1:100	ThermoFisher (Waltham, MA, USA)	A21271
GFAP	Rabbit	1:500	DAKO (Glostrup, Denmark)	Z033429-2
TH	Rabbit	1:500	Millipore (Burlington, MA, USA)	AB152
BrdU	Rat	1:100	Abcam (Cambridge, UK)	AB6326-1001
SV2	Mouse	1:1000	DSHB (Iowa City, IA, USA)	N/A
LCP1	Rabbit	1:100	Genetex (Irvine, CA, USA)	GTX124420
Tbr2a	Rabbit	1:500	Gift from the Yoshihara lab	
Secondaries: anti-mouse, rabbit, and rat IgG	Goat	1:200	Invitrogen (Waltham, MA, USA)	A11001 A11005 A11008 A11012 XH346510

## Data Availability

The data obtained in this study are available upon reasonable request to the corresponding author.

## Abbreviations

The following abbreviations are used in this manuscript:

OB	Olfactory bulb
PD	Directory of open access journals
6-OHDA	6-hydroxydopamine
DA	Dopaminergic
OSN	Olfactory Sensory Neuron
OE	Olfactory Epithelium
dpi	Days post-injection
TH	Tyrosine hydroxylase
TUNEL	dUTP Nick-End Labeling
SV2	Synaptic vesicle protein
dG	Dorsolateral glomerular cluster
dlG	Dorsolateral glomerular cluster
vmG	Ventromedial glomerular cluster
cad	Cadaverine
ON	Olfactory nerve
ONL	Olfactory nerve layer
PRAN	Pranlukast
Lcp1	Lymphocyte cytosolic protein 1
GBC	Globose basal cell
HBC	Horizontal basal cell
O.D.	Optical density
i.c.v.	Intracerebroventricular
PFA	Paraformaldehyde
IHC	Immunohistochemistry
NGS	Normal goat serum
